# The Effect of the Ketogenic Diet on Aggression and Violence in Patients with Severe Mental Illness: A Systematic Review

**DOI:** 10.1192/bjo.2025.10213

**Published:** 2025-06-20

**Authors:** Eleanor Rees, Gurpreet Kaler

**Affiliations:** 1 University of Sheffield, Sheffield, United Kingdom.; 2 Rampton Hospital, Nottingham, United Kingdom

## Abstract

**Aims:** The aim of this systematic review was to explore the existing literature on the impact of the ketogenic diet on aggressive and violent behaviour in patients with serious mental illness and the potential mechanisms involved, with the hypothesis that the ketogenic diet can reduce aggression and violence in this patient population. The ketogenic diet has proven to be useful as a therapeutic to reduce some clinical symptoms of certain neurological and psychiatric conditions, so this review was interested to determine if there were any correlations in impacts on behaviour in similar patient populations.

**Methods:** Following the PRISMA guidelines, a systematic review was conducted of the bibliographic databases MEDLINE, PsycINFO, Scopus, Web of Science, Cochrane Library, PubMed and Open Grey. The sources retrieved were narrowed down using specific inclusion and exclusion criteria and quality appraisal of the relevant sources was carried out using the Joanna Briggs Institute critical appraisal tools.

**Results:** Of the 32 sources included in the final review, 26 of these, when linked together by association, supported the concept of the ketogenic diet reducing aggression either directly or indirectly via metabolites upon which the ketogenic diet can impact. Increased β-hydroxybutyrate, γ-aminobutyric acid and brain-derived neurotrophic factor were all observed when following the ketogenic diet and were, in most cases, associated with reduced aggression.

**Conclusion:** Despite the limited literature available on the topic, the majority of the relevant sources supported the notion that the ketogenic diet could generally reduce aggression, an observation that could often be replicated in psychiatric settings. The conclusions made in this review were mostly formed by making associations between the available sources, so future research would need to be conducted with the specific focus of observing the impacts of the ketogenic diet on behaviour in psychiatric settings. Randomised controlled trials should be conducted in both inpatient and outpatient settings to enable further systematic reviews and meta-analyses to evaluate the ketogenic diet’s potential for use as a non-pharmacological therapeutic in prescribing and patient care.

